# Role of metformin in epigenetic regulation of placental mitochondrial biogenesis in maternal diabetes

**DOI:** 10.1038/s41598-020-65415-0

**Published:** 2020-05-20

**Authors:** Shaoning Jiang, April M. Teague, Jeanie B. Tryggestad, Mary E. Jensen, Steven D. Chernausek

**Affiliations:** Department of Pediatrics, Section of Diabetes and Endocrinology, Harold Hamm Diabetes Center, University of Oklahoma Health Sciences Center, Oklahoma City, OK USA

**Keywords:** Developmental biology, Endocrinology, Molecular medicine

## Abstract

Adverse maternal environments, such as diabetes and obesity, impair placental mitochondrial function, which affects fetal development and offspring long-term health. The underlying mechanisms and effective interventions to abrogate such effect remain unclear. Our previous studies demonstrated impaired mitochondrial biogenesis in male human placenta of diabetic mothers. In the present studies, epigenetic marks possibly related to mitochondrial biogenesis in placentae of women with diabetes (n = 23) and controls (n = 23) were analyzed. Effects of metformin were examined in human placental explants from a subgroup of diabetic women and in a mouse model of maternal high fat diet feeding. We found that maternal diabetes was associated with epigenetic regulation of mitochondrial biogenesis in human placenta in a fetal sex-dependent manner, including decreased histone acetylation (H3K27 acetylation) and increased promoter methylation of peroxisome proliferator-activated receptor gamma coactivator 1-alpha (PGC-1α). In male placenta, the levels of H3K27 acetylation and PGC-1α promoter methylation correlated significantly with the activity of AMP-activated protein kinase (AMPK). Metformin treatment on male diabetic placental explant activated AMPK and stimulated PGC-1α expression, concomitant with increased H3K27 acetylation and decreased PGC-1α promoter methylation. *In vivo*, we show that maternal metformin treatment along with maternal high fat diet significantly increased mouse placental abundance of PGC-1α expression and downstream mitochondrial transcription factor A (TFAM) and inhibited maternal high fat diet-impaired placental efficiency and glucose tolerance in offspring. Together, these findings suggest the capability of metformin to stimulate placental mitochondrial biogenesis and inhibit the aberrant epigenetic alterations occurring in maternal diabetes during pregnancy, conferring protective effects on offspring.

## Introduction

Diabetes during pregnancy, including gestational diabetes and pre-existing diabetes, impacts fetal growth and development and predisposes offspring to obesity, type 2 diabetes, and other metabolic diseases later in life^1–5^. The placenta is the critical organ mediating all communications between mother and fetus and is responsible for nourishing and protecting a fetus during pregnancy^6^. Intrauterine exposure to a diabetic environment can profoundly disturb the development, structure and function of placenta, in turn impacting fetal growth and increasing the risk for metabolic disease in later life^7–9^. Similar to other metabolic active tissues, placenta itself has a high energy demand and is highly susceptible to mitochondrial damage by metabolic stresses including hypoxia, obesity, and diabetes^10–12^. Placental mitochondrial defects in maternal diabetes can result in increased oxidative stress, reduced energy expenditure and impaired placental fat utilization, leading to detrimental metabolic outcomes in offspring^11,12^. However, the molecular mechanisms underlying placental mitochondrial defects in maternal diabetes remain unclear.

Epigenetic modulation of gene expression, the heritable regulation of gene activity in the absence of change in DNA sequence, plays a vital role in placental and fetal development^13^. Altered placental gene expression as an adaptive response to an adverse intrauterine environment is thought to result from epigenetic modifications^14–17^ such as DNA methylation and histone acetylation. These modifications alter DNA chromatin structure and accessibility, thereby regulating patterns of gene expression. Genome-wide DNA methylation studies on human placenta demonstrate that diabetes during pregnancy has epigenetic effects on genes involved in metabolic pathways, with consequences on fetal growth and development^18,19^. It remains unclear what specific regulatory pathways lead to such epigenetic changes in response to maternal diabetes and whether relevant epigenetic marks in maternal diabetes could be reversed or prevented.

Metformin is widely used as the first line medication for treating type 2 diabetes due to its cardiovascular protective and anti-obesity benefits, as well as low risk of hypoglycemia. However, the mechanisms underlying the actions of metformin are incompletely understood. There is evidence that metformin acts via activation of AMP-activated protein kinase (AMPK), the energy sensor and key regulator of cellular metabolism. Activated AMPK subsequently suppresses hepatic glucose production, increases peripheral glucose uptake, improves the circulating lipid profile, and reduces inflammation.

Metformin use in pregnancy remains under debate as metformin readily crosses the placenta and current knowledge about the effects of metformin on placenta and offspring remains incomplete. The Society for Maternal-Fetal Medicine (SMFM) endorsed metformin as a safe first-line pharmacologic alternative to insulin for treating gestational diabetes as available data based on neonatal outcomes support the safety of metformin use in pregnancy^20^. Following this, Loeken *et al*.^21^ published a cautionary response against the SMFM statement due to concerns regarding the possible role of metformin in inhibiting mitochondrial respiratory complex I, and affecting nutrient restriction, and epigenetic regulation, which potentially inhibit growth of placenta and fetus, and also may have long-term effects on offspring. Thus, better delineation of the short- and long-term effects of metformin is needed.

Previous studies by our groups and other groups showed impaired mitochondrial biogenesis in male placentae of mothers with diabetes^22,23^, which was associated with decreased AMPK activation^24^. The present study explores epigenetic effects of maternal diabetes and investigates the roles of metformin in epigenetic regulation of placental mitochondria biogenesis. Our findings suggest the ability of metformin to stimulate human placental mitochondrial biogenesis and reverse the altered epigenetic marks that accompany maternal diabetes.

## Results

### Clinical characteristics of the human subjects providing placenta samples

Clinical characteristics of the human subjects providing placenta samples are demonstrated in Table 1. The mothers with diabetes during pregnancy had a significantly higher HbA1C % than control subjects. Among the 23 mothers with diabetes, 16 had gestational diabetes and 7 had type 2 diabetes. Nine were treated with insulin, 2 with glyburide, and 12 with diet alone. As pre-existing type 2 diabetes and gestational diabetes constitute a similar diabetic intrauterine environment for fetoplacental development, and both maternal diabetic types increase risk of childhood obesity and type 2 diabetes in the offspring^1,25^, in the analysis of our present study, women with pre-existing type 2 diabetes and gestational diabetes are included in a single group. There is no significant difference in maternal age, body mass index, gestational age, or offspring birth weight between control and DM groups. Of note, as the patients with type 2 diabetes or gestational diabetes recruited in our study were classified as obese by BMI, the non-diabetic controls were also obese and matched for BMI in an attempt to study the isolated effects of maternal diabetes.

**Table 1:** Characteristics of Research Subjects Providing Placenta Samples Values are means ± SD. BMI: Body Mass Index (kg/m^2^).

	DM	Control	P-value DM vs Control
n	23 (16 GDM + 7 T2DM)	23	
Sex	11 male + 12 female	11 male + 12 female	
Maternal Age, yrs	32.7 +/− 5.1	30.9 +/− 5.3	0.240
Maternal HbA1c, %	5.9 +/− 1.1	5.2 +/− 0.26 (n = 11)	0.029*
Maternal BMI	34.7 +/− 8.0	30.4 +/− 5.6	0.104
Gestational age, weeks	38.7 +/− 0.9	39.1 +/− 0.5	0.086
Birth weight, kg	3.65 +/− 0.47	3.57 +/− 0.43	0.555

### Maternal diabetes decreases histone acetylation, which correlates with indices of mitochondrial biogenesis in the placenta of male offspring

**Figure 1:**
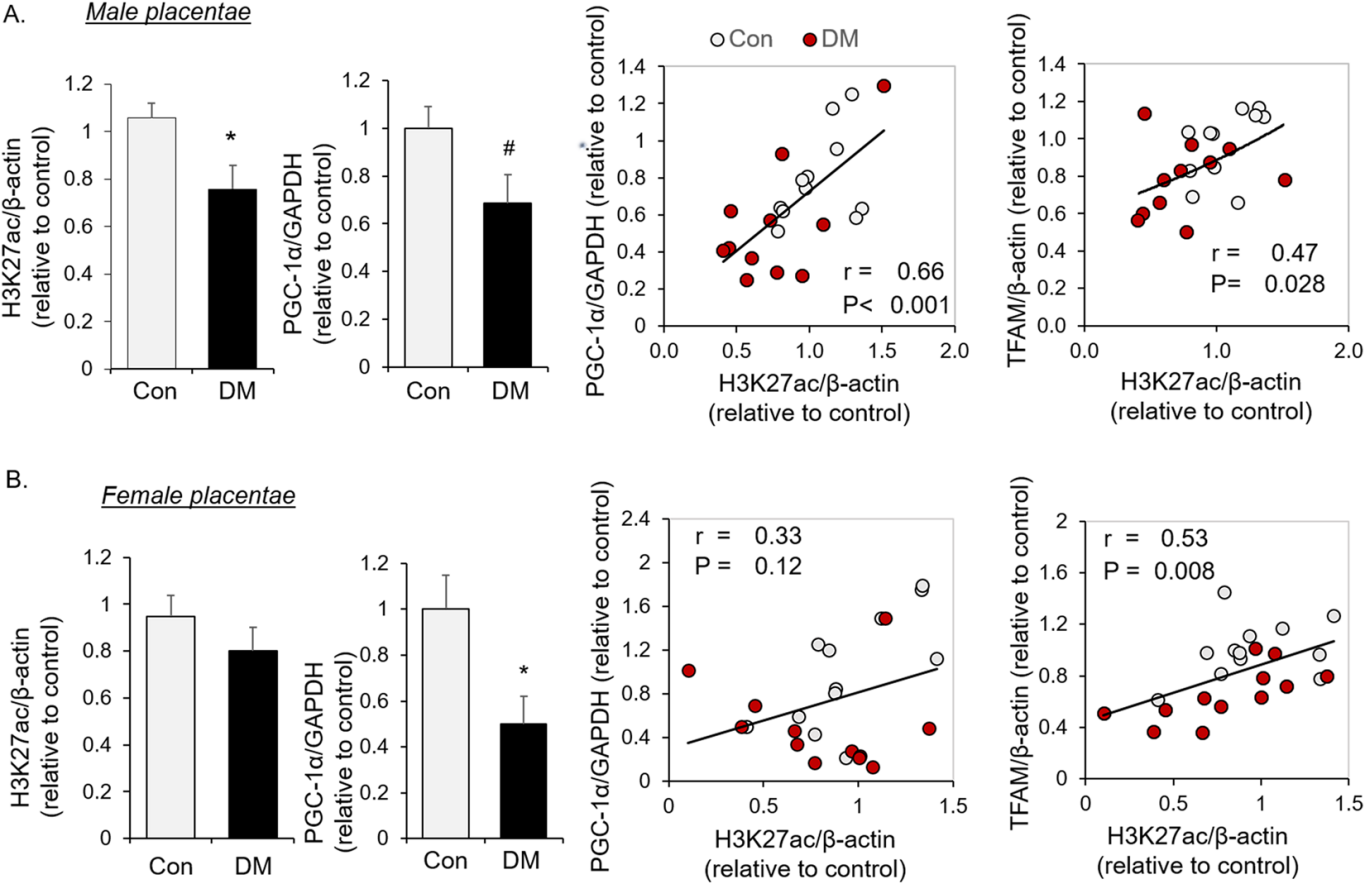
Histone acetylation and PGC-1α abundance in placental tissues of diabetic patients. Western blot quantification of H3K27ac and PGC-1α, and the correlation of H3K27ac with PGC-1α and TFAM in male human placental tissues (**A**) from diabetic patients (DM, n = 11) and healthy controls (Con, n = 11), or female placental tissues (**B**) from diabetic patients (DM, n = 12) and healthy controls (Con, n = 12) were shown. Bar graphs are presented as mean ± SEM, *P < 0.05; ^#^P < 0.05 by one-tail T-test.

In this cohort of human subjects, we demonstrated lower PGC-1α in the placenta from both male and female offspring of mothers with diabetes (Fig. 1A,B), consistent with the results of our prior report^22^. To assess epigenetic changes associated with maternal diabetes, the degree of placental histone acetylation was examined. As shown in Fig. 1A, histone 3 acetylation at lysine 27 (H3K27ac) was significantly decreased in the placentae of male, but not female, offspring from mothers with diabetes (Fig. 1B) compared to controls. In placentae of males, the level of histone acetylation (H3K27ac) was correlated with the abundance of PGC-1α protein and the downstream mitochondrial transcription factor, TFAM (Fig. 1A). In contrast H3K27ac was not significantly decreased in placentae of female offspring born to women with diabetes, despite decreased PGC-1α (Fig. 1B). Also, in females, H3K27ac was not associated with PGC-1α abundance, even though an association between the degree of H3K27ac and TFAM abundance was observed (Fig. 1B). These results indicate sex-dimorphism in the regulation of histone acetylation, which likely affects placental mitochondrial biogenesis signaling in a fetal sex-dependent manner.

### Maternal diabetes increases PGC-1α promoter methylation in placentae of male offspring

**Figure 2:**
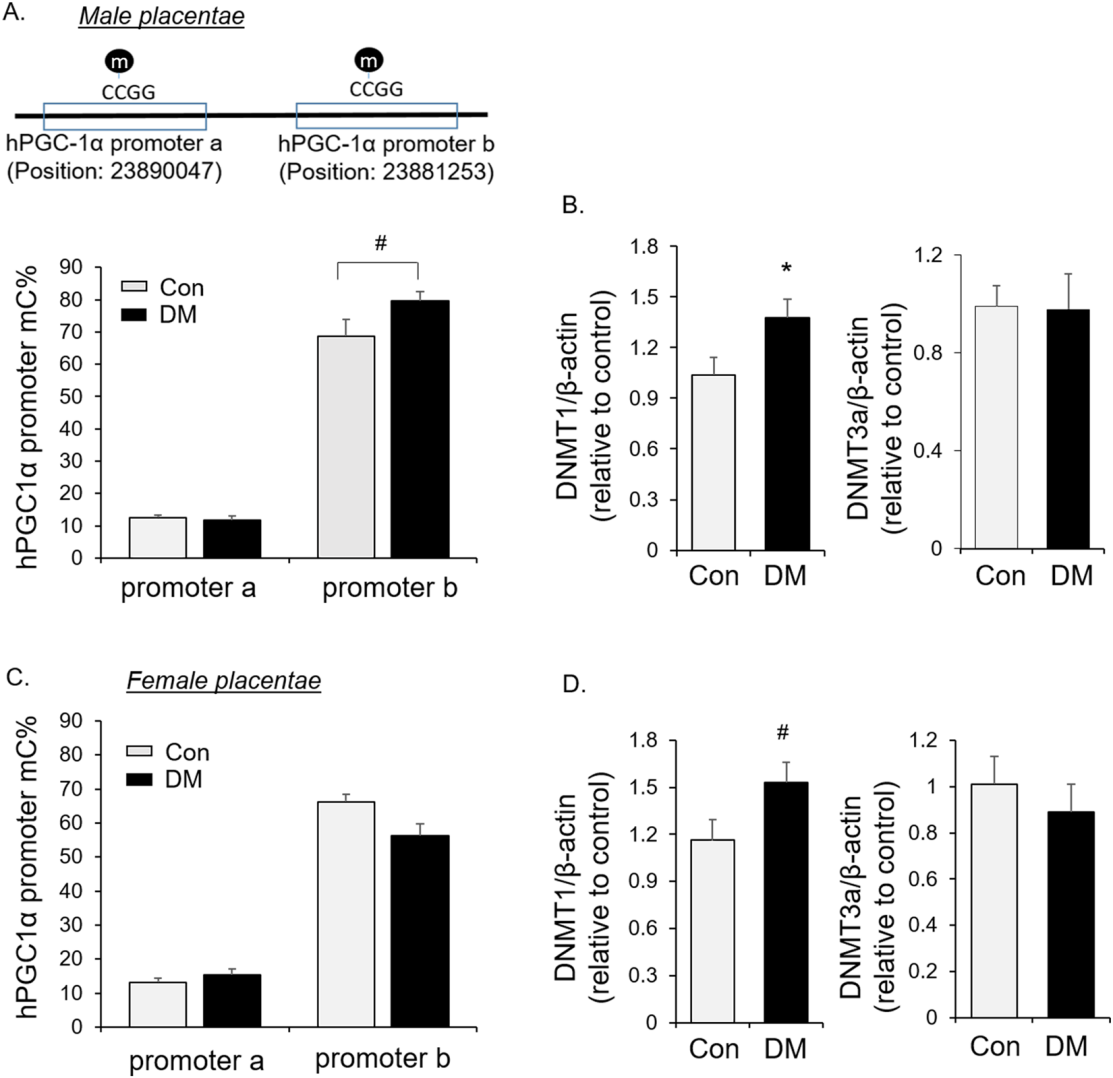
PGC-1α promoter methylation in placental tissues of diabetic patients. (**A,B**) Levels of DNA methylation on two indicated loci of PGC-1α promoters (**A**) and protein abundance of DNMT1 and 3a by Western blot analysis (**B**) in male human placentae (n = 11 in each DM and Con group); (**C,D**) Levels of DNA methylation on two indicated loci of PGC-1α promoters (**C**) and protein abundance of DNMT1 and 3a by Western blot analysis (**D**) in female human placentae (n = 12 in each DM and Con group). Bar graphs are presented as mean ± SEM, *P < 0.05; ^#^P < 0.05 by one-tail T-test.

DNA methylation is another component of epigenetic regulation which can negatively regulate PGC-1α expression^26^. PGC-1α promoter b methylation, but not promoter a, was increased in placentae of males born to mothers with diabetes compared to controls (Fig. 2A). This translates to an approximately 30% decrease of unmethylated DNA at promoter b. In addition, protein abundance of DNA methylation transferase 1 (DNMT1), but not DNMT3a, was higher in male diabetic placentae than controls (Fig. 2B). No increase in methylation of either promoter of PGC-1α was observed in female placentae, despite increased DNMT1 (Fig. 2C,D). Those alterations in epigenetic marks with mitochondrial biogenesis signaling suggest a potential involvement of fetal sex-dependent epigenetic regulation in placental mitochondrial biogenesis in maternal diabetes.

### Association of AMPK activation with histone acetylation and PGC-1α promoter methylation

**Figure 3:**
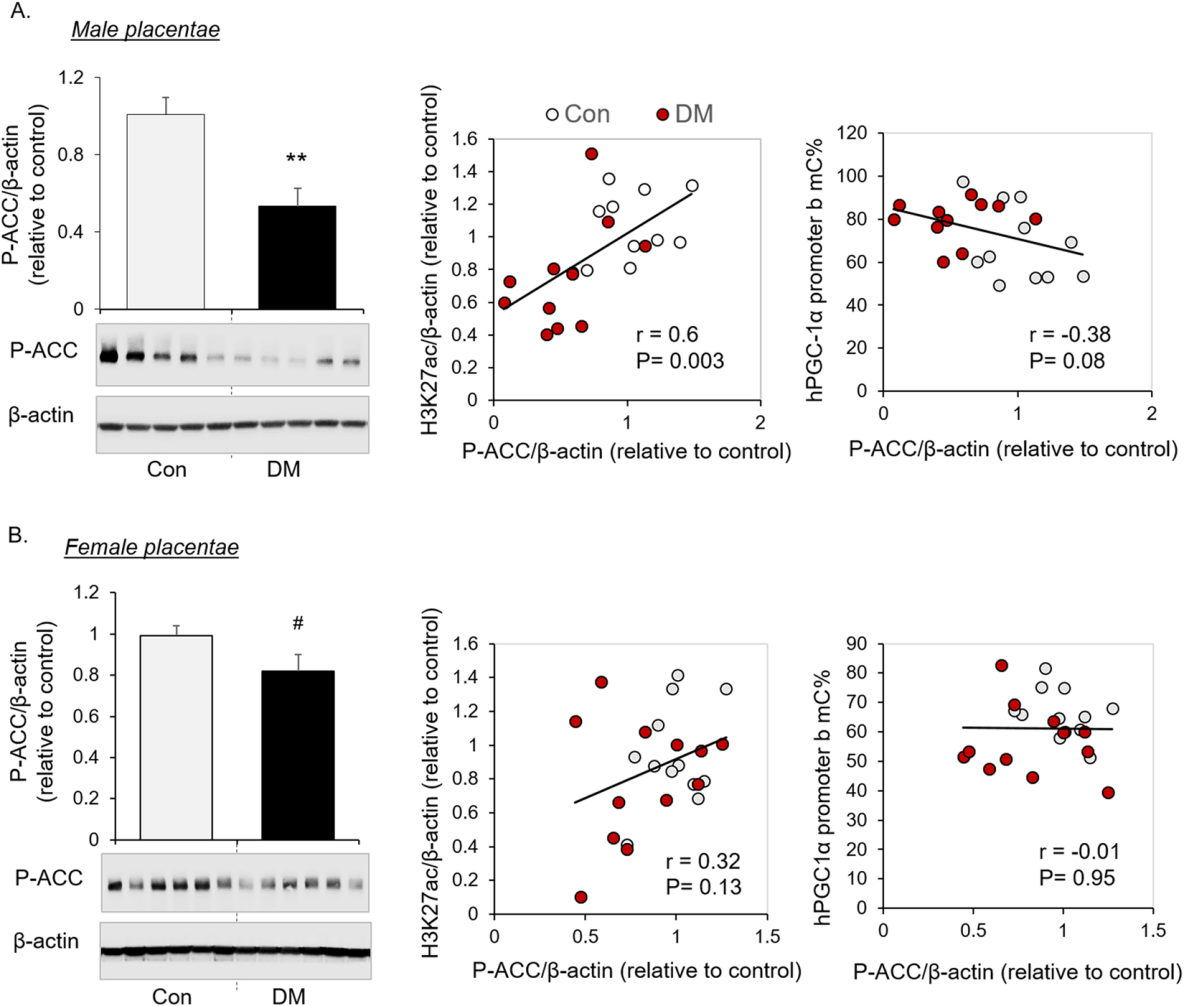
Correlation of AMPK activation with epigenetic events in human placentae. Representative cropped Western blots and quantification of P-ACC, and the correlation of P-ACC with H3K27ac and PGC-1α methylation of promoter b in male human placentae (**A**, n = 11 in each DM and Con group) or female human placentae (**B**, n = 12 in each DM and Con group) are shown. Bar graphs are presented as mean ± SEM, **P < 0.01; ^#^P < 0.05 by one-tail T-test. Full-length blots are provided in the Supplementary information.

Recent evidence demonstrates a role of AMPK in regulating epigenetic modifications. We found that AMPK was inhibited in placentae of diabetic mothers, evidenced by decreased Phospho-Serine79-Acetyl-CoA Carboxylase (P-ACC), a surrogate marker of AMPK activation (Fig. 3A,B, Full-length blots are provided in the Supplementary information). The level of P-ACC was positively associated with H3K27 acetylation and approaching negative association with PGC-1α promoter methylation in male placentae (Fig. 3A), but not in female placentae (Fig. 3B), suggesting a potential participation of AMPK in regulating H3K27 acetylation in maternal diabetes.

### Metformin treatment stimulated AMPK/PGC-1α signaling and altered the relevant epigenetic modifications in human placental explants

**Figure 4:**
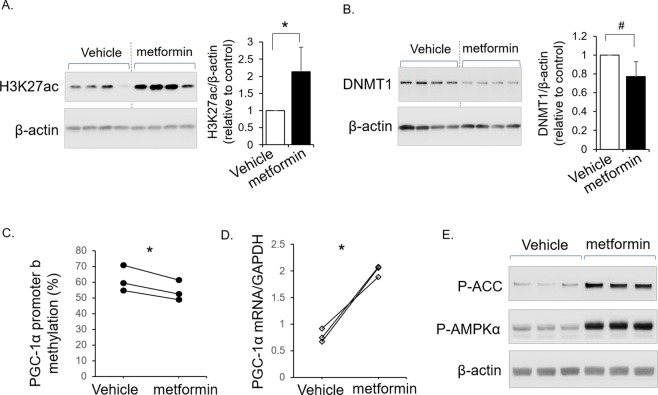
Metformin increased histone acetylation and decreased DNMT1 and PGC-1α promoter methylation in human placental explants, concomitant with mitochondrial biogenesis signaling. Male placental explants from diabetic patients (n = 4 patients) were treated with metformin (1 mg/ml) or vehicle (H_2_O) for 18 hours in culture (plated in 4 wells in each group). (**A,B**) Levels of H3K27ac (**A**) and DNMT1 (**B**) were determined by Western blot analysis. Representation blots and bar graph of average fold changes in all male placental explants (n = 4 explants from different patients) were shown. (**C,D**) Placental explants with metformin treatment or vehicle controls were subjected for DNA methylation analysis and RNA extraction, and average of PGC-1α methylation of promoter b (**C**) and PGC-1α mRNA expression (**D**) in each individual placental explant were determined by RT-PCR (n = 3 explants from different patients). (**E**) Representation blots of P-ACC and P-AMPKα were shown. Mean ± SD, *P < 0.05 by paired T-test; ^#^P < 0.05 by one-tail paired T-test. Full-length blots are provided in the Supplementary information.

As metformin is a known activator of AMPK, we next determined whether metformin can reverse the altered epigenetic marks and mitochondrial biogenesis in maternal diabetes. Placental explants from a subgroup of mothers with diabetes were cultured and treated with metformin. Metformin significantly increased the level of histone acetylation (H3K27ac) in the human placental explants from male offspring (Fig. 4A). Metformin treatment also resulted in decreased protein abundance of DNMT1 (Fig. 4B). DNA methylation analysis on male placental explants showed decreased PGC-1α promoter methylation after metformin treatment (Fig. 4C), paralleled with increased PGC-1α mRNA expression (Fig. 4D). Metformin potently activated AMPK in diabetic placentae, demonstrated by increased ACC and AMPK phosphorylation (Fig. 4E). These results suggest the capability of metformin to attenuate the mitochondrial biogenesis-related epigenetic modifications as observed in male placentae of diabetic mothers and to stimulate AMPK/PGC-1α mitochondrial biogenesis signaling. Results from the 2 female placental explants showed similar changes by metformin in H3K27ac and DNMT1 (data not shown), but remain to be further evaluated in future studies by including more individuals with female offspring.

### Metformin treatment in mice improved male placental efficiency, associated with increased placental TFAM and decreased PGC-1α promoter methylation

**Figure 5:**
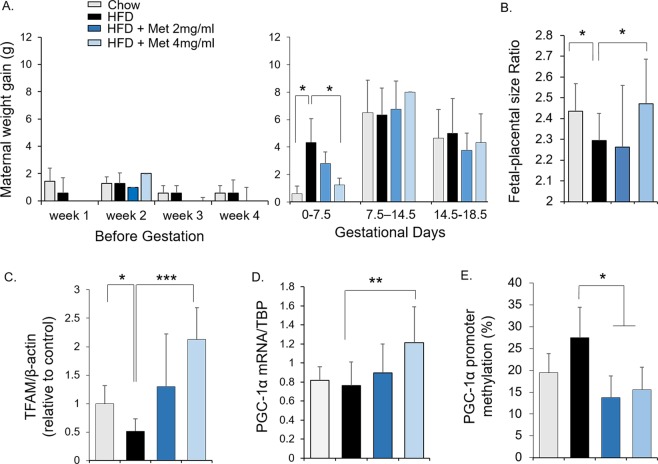
*In vivo* effects of metformin on placenta. Mice were fed either with a control diet (chow), or a high-fat diet (HFD), or HFD with metformin (2 mg/ml or 4 mg/ml) from 6–8 weeks before mating and throughout gestation. (**A**) Maternal weight gain before gestation and during different gestational periods. (**B**) Fetal-placental size ratios. (**C–E**) TFAM protein abundance (**C**), PGC-1α mRNA expression (**D**), and PGC-1α promoter methylation (**E**) in male placentae. Bar graphs were presented as mean ± SD, *P < 0.05; ***P < 0.001. Chow: n = 6–9; HFD: n = 9–14; HFD + Met (2 mg/ml): n = 5; HFD + Met (4 mg/ml): n = 4–5.

To examine the effects of metformin *in vivo*, female mice were fed with chow diet, high fat diet or high fat diet in combination with metformin 6-8 weeks before mating and sacrificed at 18.5 days of gestation. Before gestation, weight gain in mice did not differ between the groups, whereas maternal weight gain was significantly greater in mice on high fat diet than on chow diet during the first week of gestation, which was prevented by metformin treatment at the higher dose (4 mg/ml) (Fig. 5A). Compared to chow diet-fed mice, the fetal-placental size ratio was decreased in mice with maternal high fat diet, but not in high fat diet-fed mice with metformin treatment (dose of 4 mg/ml, Fig. 5B), suggesting a role of metformin in improving placental efficiency. Maternal high fat diet was associated with reduced placental TFAM expression which was prevented by metformin treatment dose-dependently (Fig. 5C). Consistent with what observed in human placental explant (Fig. 4C), maternal metformin treatment resulted in decreased DNA methylation on PGC-1α promoter in male placentae of mice fed with high fat diet (Fig. 5E), which was associated with increased PGC-1α expression (Fig. 5D).

### Maternal metformin treatment improved male offspring glucose homeostasis in response to maternal high fat diet feeding

**Figure 6:**
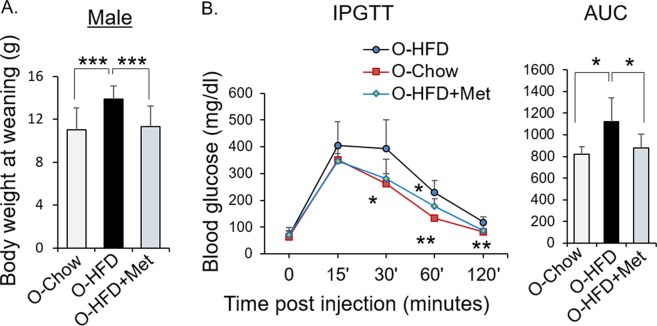
Effects of metformin on offspring. Mice were fed either with Chow, or HFD, or HFD with metformin (4 mg/ml) from 6-8 weeks before mating, throughout gestation and lactation. Offspring were studied at weaning (day 23). (**A**) Body weight of male offspring. O-Chow: n = 19; O-HFD: n = 17; O-HFD + Met (4 mg/ml): n = 19. (**B**) Glucose tolerance of male offspring. Blood glucose levels during the glucose tolerance test and the area under the curve. O-Chow: n = 5; O-HFD: n = 5; O-HFD + Met (4 mg/ml): n = 6. Mean ± SD, *P < 0.05; **P < 0.01; ***P < 0.001.

To evaluate the effects of maternal metformin treatment on the offspring, another set of dams were fed with either chow diet, high fat diet, or high fat diet with metformin. The body weight of the male offspring of HFD-fed dams, but not HFD with metformin-treated dams, at weaning were significantly higher than chow diet offspring (Fig. 6A). Maternal high fat diet resulted in impaired glucose tolerance in the offspring, which was prevented by metformin treatment (Fig. 6B). Together, maternal metformin treatment improved placental efficiency and offspring glucose homeostasis in response to maternal high fat diet feeding.

## Discussion

**Figure 7:**
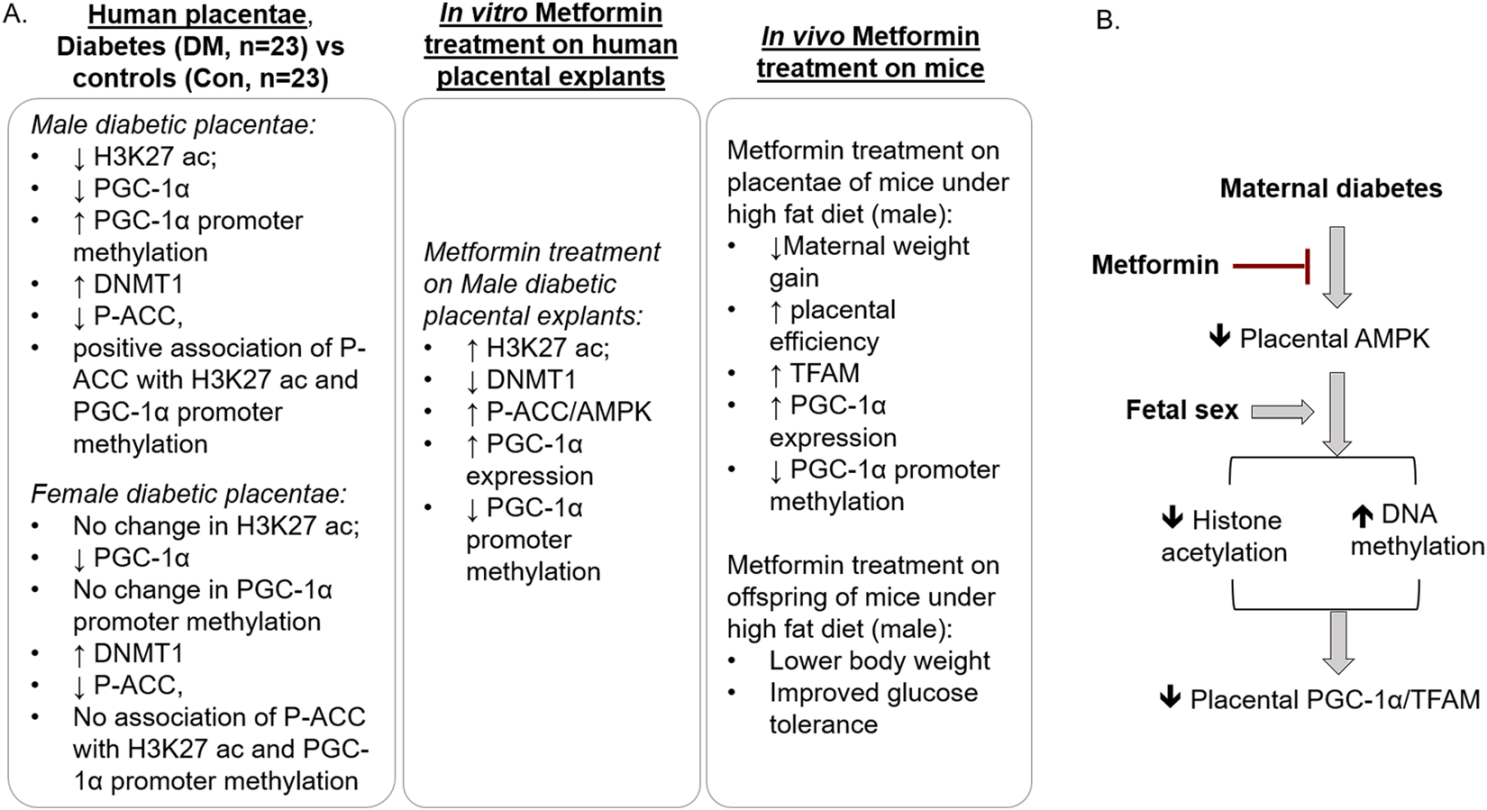
Summarized diagram. (**A**) Diagram of main findings in each experimental settings. (**B**) Summarized diagram. Maternal diabetes inhibits placental AMPK activity, which is associated with epigenetic regulation of PGC-1α/TFAM signaling in a fetal sex-dependent manner. Metformin treatment activates placental AMPK, stimulates placental mitochondrial biogenesis and inhibits the aberrant epigenetic alterations occurring in maternal diabetes during pregnancy.

The present study extends our previous findings on the effects of maternal diabetes on human placental PGC-1α and downstream mitochondrial biogenesis and content^22^ and reveals that diabetes during pregnancy results in alterations in epigenetic marks and AMPK activity that correlate with changes in pathways regulating mitochondrial biogenesis in a fetal-sex dependent manner (Fig. 7). Utilizing human placental and animal models, we further demonstrate that metformin treatment inhibits those epigenetic alterations, stimulates placental mitochondrial biogenesis, and improves offspring glucose tolerance.

Functional mitochondria are essential for placental metabolism, growth, steroid synthesis, and active nutrient transport^10,27^. We previously reported that PGC-1α and the activity of the nutrient and energy sensor AMPK were decreased in placentae from Native American and Hispanic women with diabetes during pregnancy^22,24^. The present study analyzed placentae collected from newly-recruited subjects of any ethnic background after delivery by elective Cesarean section. Consistent decreases in AMPK activity and PGC-1α protein were observed in placentae of both sexes, confirming the effects of diabetes during pregnancy in placenta regardless of ethnicity and mode of delivery. Since AMPK stimulates mitochondrial biogenesis by increasing expression and activation of PGC-1α^28^, the decreased placental AMPK activity is likely an important contributor to impaired placental mitochondrial biogenesis in maternal diabetes.

Epigenetic modifications are key determinants of important developmental events during fetal life, and emerging evidence identifies AMPK as an important epigenetic regulator. AMPK activation can induce histone acetylation by activating histone acetyltransferase 1 (HAT1) as well as increasing production of acetyl-coA, a substrate of histone acetyltransferase^29^. Here we identified alteration of a specific histone acetylation site, histone 3 lysine 27 (H3K27 acetylation), which was decreased in male human placentae of diabetic patients. H3K27 acetylation is thought to be a critical element in fetal developmental^30^ and is reported to be tightly coupled to epigenetic regulation of transcription factors involved in the pathology of fetal growth restriction^14^. The significant correlation among levels of AMPK activation, H3K27 acetylation and PGC-1α/TFAM signaling in male human placentae, suggests a potential effect of AMPK on H3K27 acetylation and subsequent mitochondrial biogenesis. The association of maternal diabetes with histone acetylation was significant in placentae of males but not females, indicating an effect of fetal sex on epigenetic responses to placental AMPK activation and mitochondrial biogenesis.

PGC-1α promoter activity is tissue-specific^31^, and transcriptional regulation of PGC-1α by DNA methylation has been shown to be CpG site-specific in multiple human tissues, including placenta and skeletal muscle^32,33^. The extent to which promoter activity is regulated by DNA methylation in human placentae is unknown. Here we examined methylation of CpG sites of two PGC-1α promoters. We found the CpG site in PGC-1α promoter b to be highly methylated, which was accentuated in male placenta of diabetic mothers. DNMT1 is the predominant mammalian DNA methylating enzyme responsible for maintenance of DNA methylation, typically inhibiting gene transcription. Maternal diabetes is reported to be associated with increased global DNA methylation in placentae^34^. We found that the abundance of DNMT1, but not DNMT3a was increased in human placentae of diabetic mothers, which might explain the increased placental DNA methylation. PGC-1α DNA methylation was increased in placentae from males, but not females, born to mothers with diabetes, further demonstrating sexual dimorphism in placental epigenetic regulation of mitochondrial biogenesis in response to maternal diabetes. Decreased PGC-1α protein in female placentae of diabetic women is likely independent of H3K27 acetylation and promoter DNA methylation. Other mechanisms, such as decreased activity of PGC-1α transcription factors, including FoxO1, ATF2, and USF-1 etc, can result in decreased PGC-1α expression^35,36^.

Metformin is a potent AMPK activator, but knowledge of the effects of metformin on the placenta are limited. Human studies showed that metformin treatment of patients with gestational diabetes produced beneficial effects on placental morphology^37^. Here, we found metformin treatment on male placental explants from diabetic women resulted in AMPK activation and increased expression of PGC-1α, indicating the potential for metformin to reverse maternal diabetes-induced impairment in placental mitochondrial biogenesis. Similar stimulation of mitochondrial biogenesis signaling was induced by another AMPK activator AICAR (data not shown), suggesting that AMPK is likely mediating such effects. In addition to stimulating mitochondrial biogenesis, AMPK activation can also exert anti-inflammatory effect and can promote mitophagy^38,39^, resulting in improved mitochondrial quality, decreased inflammation and increased cell survival, which can explain the beneficial effects of metformin on placenta.

Increased H3K27 acetylation is reported to be associated with enhanced expression of transcription factors involved in mitochondrial biogenesis, including PGC-1α, nuclear respiratory factor 1, and TFAM^40,41^. Exposure of male placental explants from mothers with diabetes to metformin increased H3K27 acetylation and PGC-1α/TFAM signaling, thus correcting the aberrant H3K27 acetylation in maternal diabetes and, consequently, expression of genes involved in placental mitochondrial biogenesis. In addition, metformin treatment significantly decreased the level of DNMT1, concomitant with decreased DNA methylation of PGC-1α promoter b and increased PGC-1α expression. Consistent with these results, metformin treatment in a murine model decreased placental PGC-1α promoter methylation and increased mRNA level of PGC-1α. Therefore, metformin plays a role in epigenetic regulation of placental mitochondrial biogenesis, likely involving both components of epigenetic modifications: DNA methylation and histone acetylation.

The placenta plays an important role in modulating fetal responses to an adverse maternal metabolic environment, and interventions that improve placental function may benefit the fetus and improve offspring long-term health^42^. A recent randomized trial showed that metformin during pregnancy could reduce premature birth and miscarriage in women with polycystic ovary syndrome (PCOS)^43^. Similarly, preventing maternal diabetes-induced reduction in placental AMPK activity and mitochondrial biogenesis with metformin might protect the metabolic status of the offspring. This is supported by our results in mice in which maternal metformin treatment prevented the high fat diet-induced impairment in placental efficiency and offspring glucose tolerance at weaning. Of note, there appears a dose-dependent effect of metformin in mice, in which the improved placental efficiency and elevated PGC-1α and TFAM expression were only achieved by the higher dose of metformin provided in drinking water (4 mg/ml).

Previous studies reported more pronounced decreases in placental PGC-1α expression and AMPK activation in GDM treated by insulin/glyburide treatment compared to dietary intervention^11^. We compared the levels of placental PGC-1α, H3K27ac, and DNMT1 between insulin/glyburide and diet treated pregnancies in each fetal sex, and found no significant difference (data not shown). Considering the sample size (n = 4 diet in male, n = 4 insulin/glyburide in female) seems too small, the effects of medical interventions remain to be further evaluated in future studies.

Maternal high fat diet mouse model has been widely used as an animal model to study the developmental programming of offspring metabolic diseases^44^. We demonstrated that maternal high fat diet resulted in decreased placental efficiency, decreased placental mitochondrial biogenesis signaling, and impaired glucose tolerance in the offspring, recapitulating some of the findings in humans exposed to diabetes during pregnancy. It is therefore a suitable animal model to study the *in vivo* effects of metformin on placenta and offspring. In our animal experiment, metformin was given in parallel with high fat diet pre-gestational and throughout gestation to demonstrate the effects of metformin on offspring in a controlled manner not easily done in humans. While metformin is not used across gestation in most clinics, therefore as a limitation, our animal studies may not give an adequate representation of the clinical standard of care. Thus, the extent to which the results of these animal studies will apply to humans is important and will entail much further study beyond the scope of what is presented here.

The strengths of our present study are identification of mitochondrial biogenesis-related epigenetic modifications associated with maternal diabetes in human placenta and investigation of the effects of metformin in both human placenta and mouse models. The altered epigenetic modifications can mediate the long-term programming effects of metformin on offspring which awaits future studies. Limitations of the present study are that the specific therapeutic window of metformin and detailed mechanisms underlying AMPK effects on epigenetic regulation of mitochondrial biogenesis remain to be explored.

Controversy regarding metformin use during pregnancy is likely to persist. The body weight of children born to mothers who received metformin during pregnancy is increased compared to those unexposed^45,46^, suggesting that they may be at increased risk for obesity and metabolic syndrome later in life. However, more direct measures of adiposity (e.g. DEXA scan) appear to be unaffected as are many other health measures^45,46^, so the impact of the relatively small increase in body weight on health remains unclear. Because of its ease of use and relative safety, as well as the role played in improving fertility in women with PCOS, it is highly likely that metformin will be continued to be used during pregnancy.

In conclusion, the present studies suggest that maternal diabetes during pregnancy results in epigenetic changes in human placenta, which correlate with indices of impaired mitochondrial biogenesis in a fetal sex-dependent manner (Fig. 7). Metformin treatment stimulated epigenetic regulation of placental mitochondrial biogenesis and improved offspring glucose homeostasis. Our data suggest that more studies evaluating potential beneficial effects of metformin are indicated and that such benefits might depend on offspring sex.

## Material and methods

### Subjects for placenta samples

Placental samples were obtained from women with gestational diabetes (GDM), pregestational type 2 diabetes, or with normoglycemic pregnancies after delivery by elective Cesarean section at term. Gestational and type 2 diabetes in the mothers was diagnosed according to ADA guidelines^47^. Women were excluded if the infants were small for gestational age, had a major malformation, or chromosome abnormality. They also were excluded if they delivered prior to 37 weeks gestation, had type 1 diabetes, pre-eclampsia, chronic hypertension, renal disorders or a smoking history of more than 5 cigarettes per day during pregnancy. The protocol was approved by the Institutional Review Board of the University of Oklahoma Health Science Center and conducted in accordance with the Declaration of Helsinki. Written informed consent was provided by all participants at enrollment. Placental tissues from 23 controls and 23 women with diabetes (DM group) with matched maternal age, maternal BMI, gestational age, and birth weight were analyzed.

### Placentae dissection

Term placentae were dissected as soon as possible after delivery, generally within 15 minutes, and processed as previously described with modifications^48^. Five samples were taken from areas evenly distributed around the circumference of the fetal side of the placenta, roughly halfway between the cord insertion site and the placental margin. Thus, the fetal part, including chorionic villi and chorionic plate, was collected as previously described^49^. Each sample was approximately 2 cm in diameter and extended approximately 1 cm below the fetal membrane. The fetal membrane was trimmed away and the remaining tissue was rinsed in cold DPBS, blotted dry, wrapped in aluminum foil and submerged in liquid nitrogen. The placental samples from five areas were combined, powered, and stored at −80 °C until analysis. For explant studies, two pieces of specimen were similarly collected, but were stored in cold DPBS until it was transported back to the lab.

### Placental explant culture

The placental explant culture was performed on a subgroup of diabetic women (including 4 male and 2 female placentae). The placental tissues were stripped of connective tissues and dissected to small pieces (about 2 mm). The placental villous explants were cultured in 12-well plate at 37 °C in 5% CO_2_ in Ham’s F-12 medium (Gibco/Life Technologies, Grand Island, NY) supplemented with 10% FBS (Mediatech, Manassas, VA), 100 µM MEM Non-Essential Amino Acids (Gibco/Life Technologies, Grand Island, NY), and 0.5% penicillin/streptomycin/amphotericin B (Gibco/Life Technologies, Grand Island, NY) and were treated with metformin (1 mg/ml, n = 4 wells) or vehicle (H_2_O, n = 4 wells) for 18 hours in culture, followed by protein analysis with Western blot and DNA/RNA analysis with quantitative RT-PCR. The average of the parameters measured in each group were calculated. Each experiment was repeated in placental explants from different patients and statistical analysis was performed on male placental explants (n = 3-4 patients depending on the availability of protein and DNA/RNA samples).

### Western Blot Analysis

Western blot analysis was performed as previously described^22^. Placental samples were lysed in RIPA buffer in the presence of protease and phosphatase inhibitor cocktail (Pierce Biotechnology, Rockford, IL). Protein concentrations were determined by BCA assay (Pierce, Rockford, IL). Thirty µg of protein lysate was reduced (Dithiothreitol, DTT), and subjected to sodium dodecyl sulfated polyacrylamide gel electrophoresis (SDS-PAGE) and transferred to polyvinylidene fluoride (PVDF) membrane. The blots were incubated with antibodies specific for Acel-K27-His3, DNMT1, DNMT3a, PGC-1α (Abcam, Cambridge, MA), TFAM, P(S97)-ACC, or β-actin (Cell Signaling Technology, Danvers, MA), and detected by enhanced chemiluminescence (Pierce, Rockford, IL). The blots were quantified and analyzed by imaging densitometry with Image Lab Software (Bio-Rad, Hercules, CA).

### DNA methylation analysis

Methylated DNA was analyzed with the EpiJET DNA Methylation Analysis kit (Msp1/Hpa11) by ThermoFisher Scientific (catalog #K1441) according to the manufacturer’s guidelines with modification. The proportion of DNA methylation of “promoter a” (as indicated in Fig. 2) of human PGC-1α was quantified by qPCR using primers as previously described^50^: Forward: AAAACGCAAACTACACAACCC, Reverse: AGGCTCCCAGAAAACAAGTG. The proportion of DNA methylation of “promoter b” of human PGC-1α was quantified using primers: Forward: CGAGGCAAAGGGTAAAGTCATA, Reverse: GAAGGTGAGTGCAGGTGAAA. The proportion of DNA methylation of the promoter of murine PGC-1α were quantified using primers: Forward: CCACAGAACACAAAACGACAG, Reverse: AAACACGCTGAAGTCCTCTG.

### mRNA quantification

Total RNA was purified from placental tissues by RNA isolation kit (miRNeasy, Qiagen) and converted to complementary DNA (cDNA) with SuperScript VILO cDNA Synthesis Kit (Invitrogen) according to the manufacturer’s instructions. Real-time qPCR was performed using TaqMan Real-Time PCR Probes for human PGC-1α or GAPDH (Life Technologies). Mouse PGC-1α mRNA were quantified using primers: Forward: CACCAAACCCACAGAAAACAG, Reverse: GTACAACTCAGATTGCTCGGG. Results were calculated using the 2^−ΔΔCt^ method normalized to GAPDH or TATA-binding protein (TBP).

### Animal experiments

C57BL/6 J mice (Jackson laboratory, USA) were maintained according to the standard protocols approved by the University of Oklahoma Animal Use and Care Committee. All experiments were performed in accordance with the relevant regulations and guidelines approved by the University of Oklahoma Institutional Animal Care and Use Committee. Mice were housed under a 12-h light/dark cycle (from 6 am to 6 pm), with ad libitum access to diet and water. At 5 weeks of age, females were randomized to receive either a control diet (D12450H, Research Diets, Inc., New Brunswick, NJ) with 10% energy from fat (Control), or a high-fat diet (HFD; D12451, Research Diets, Inc.) with 45% energy from fat with matched sucrose content, or a high fat diet with metformin supplied in drinking water (2 mg/ml or 4 mg/ml) from 6-8 weeks before mating, throughout gestation and lactation. For murine placental studies, tissues were collected at Gestation day 18.5. The sex of the fetus was determined by PCR using a primer pair (F: GATGATTTGAGTGGAAATGTGAGGTA; R: CTTATGTTTATAGGCATGCACCATGTA) that amplifies fragments from the X and Y chromosome with size difference between the amplicons as described previously^51^. The fetal-placental ratio was calculated by dividing the crown-rump length to the diameter of placenta. For offspring study, the litter size was controlled to 6 mice per litter and glucose tolerance test and body weight were measured at weaning.

### Glucose tolerance test

Offspring underwent glucose tolerance tests at weaning. The mice were fasted overnight (16 h) in fresh cages with ad libitum water supply. Body weight and fasting glucose were measured, followed by intraperitoneal injection of D-glucose (2 mg glucose/g body weight). Blood glucose levels were measured at baseline and15, 30, 60, 90 and 120 min after injection.

### Statistical methods

Differences in characteristics between two groups were assessed using Student’s t-test for continuous measures. The data sets were tested for equality of variances using Levene’s test. Parameters measured in human placenta explants in response to metformin treatment were compared by a paired t-test (n = 3-4 patients). Correlations were calculated as standardized regression coefficients. Data analyses used Excel. P-values <0.05 were treated as statistically significant for the purposes of discussion.

## Supplementary information

Supplementary information.
