# Supplementary material for: Role of metformin in epigenetic regulation of placental mitochondrial biogenesis in maternal diabetes
Source: Sci Rep. 2020 May 20;10:8314. doi: 10.1038/s41598-020-65415-0 (PMC7239922; doi:10.1038/s41598-020-65415-0)

Supplementary information

Title: **Role of metformin in epigenetic regulation of placental mitochondrial biogenesis in maternal diabetes**

Shaoning Jiang[1*], April M. Teague[1], Jeanie B. Tryggestad[1], Mary E. Jensen[1,] and Steven D. Chernausek[1]

[1]Department of Pediatrics, Section of Diabetes and Endocrinology, Harold Hamm Diabetes Center, University of Oklahoma Health Sciences Center, Oklahoma City, OK

Full-length blots for **Fig. 3A**

P-ACC

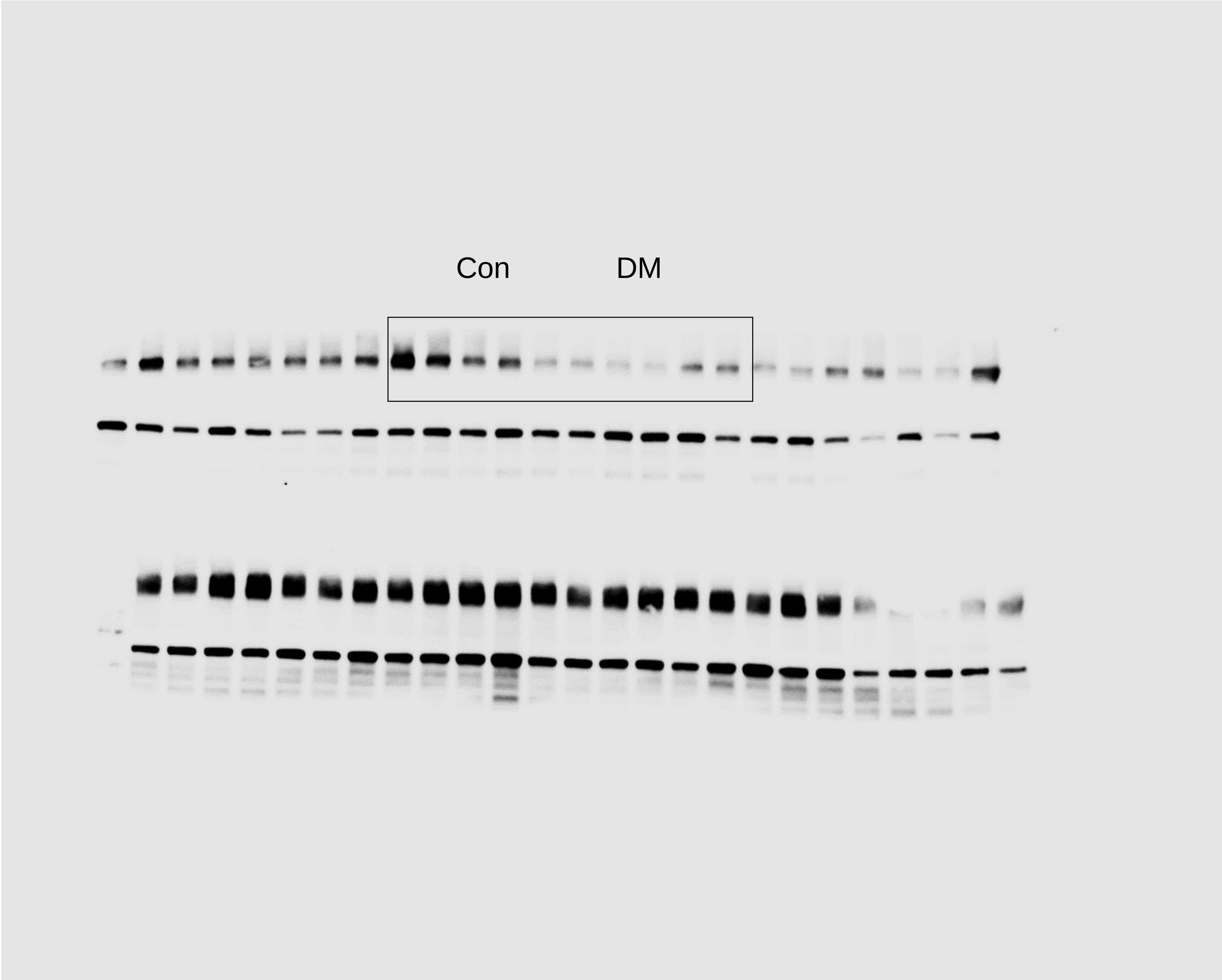

β-actin

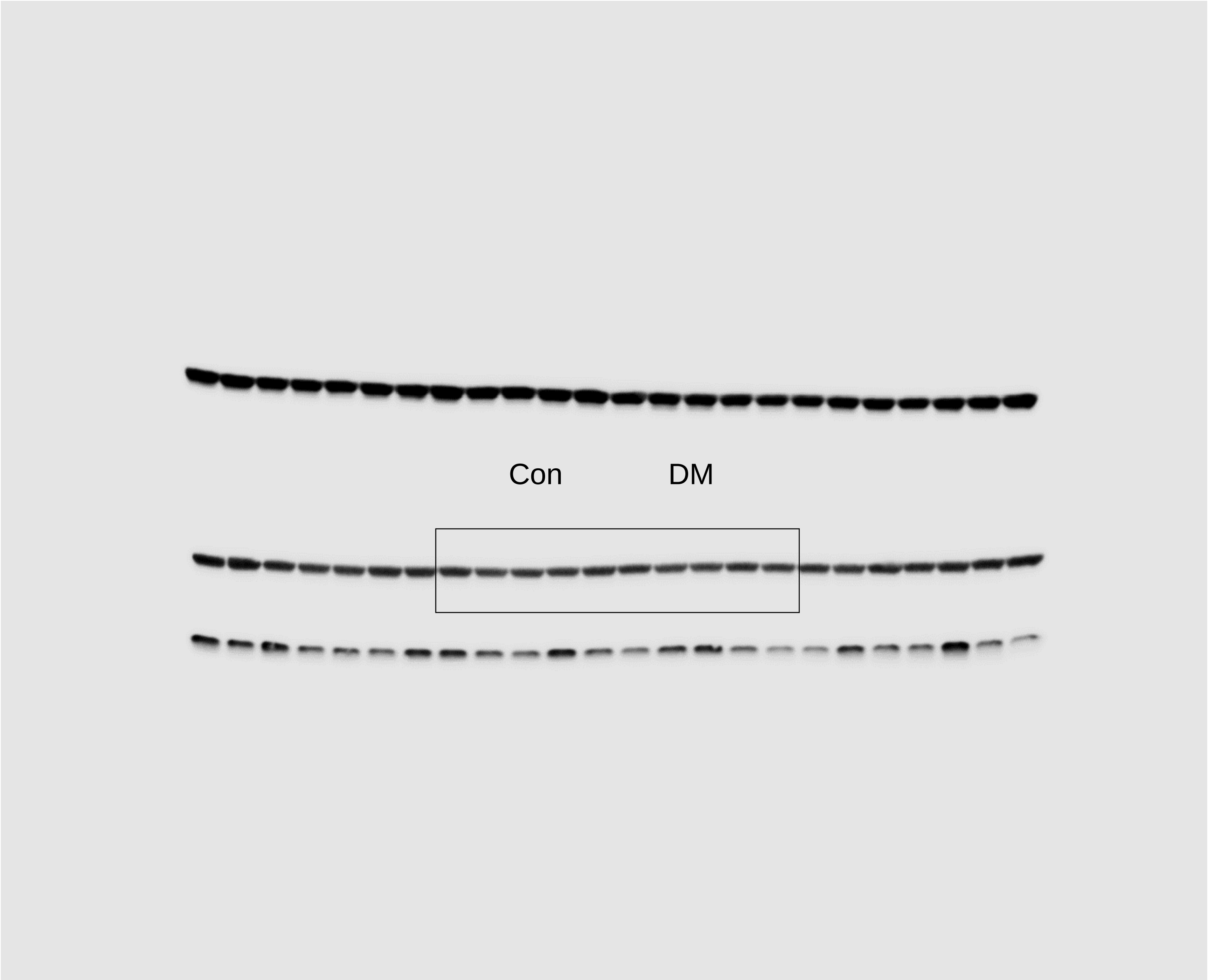

Full-length blots for **Fig. 3** B.

P-ACC

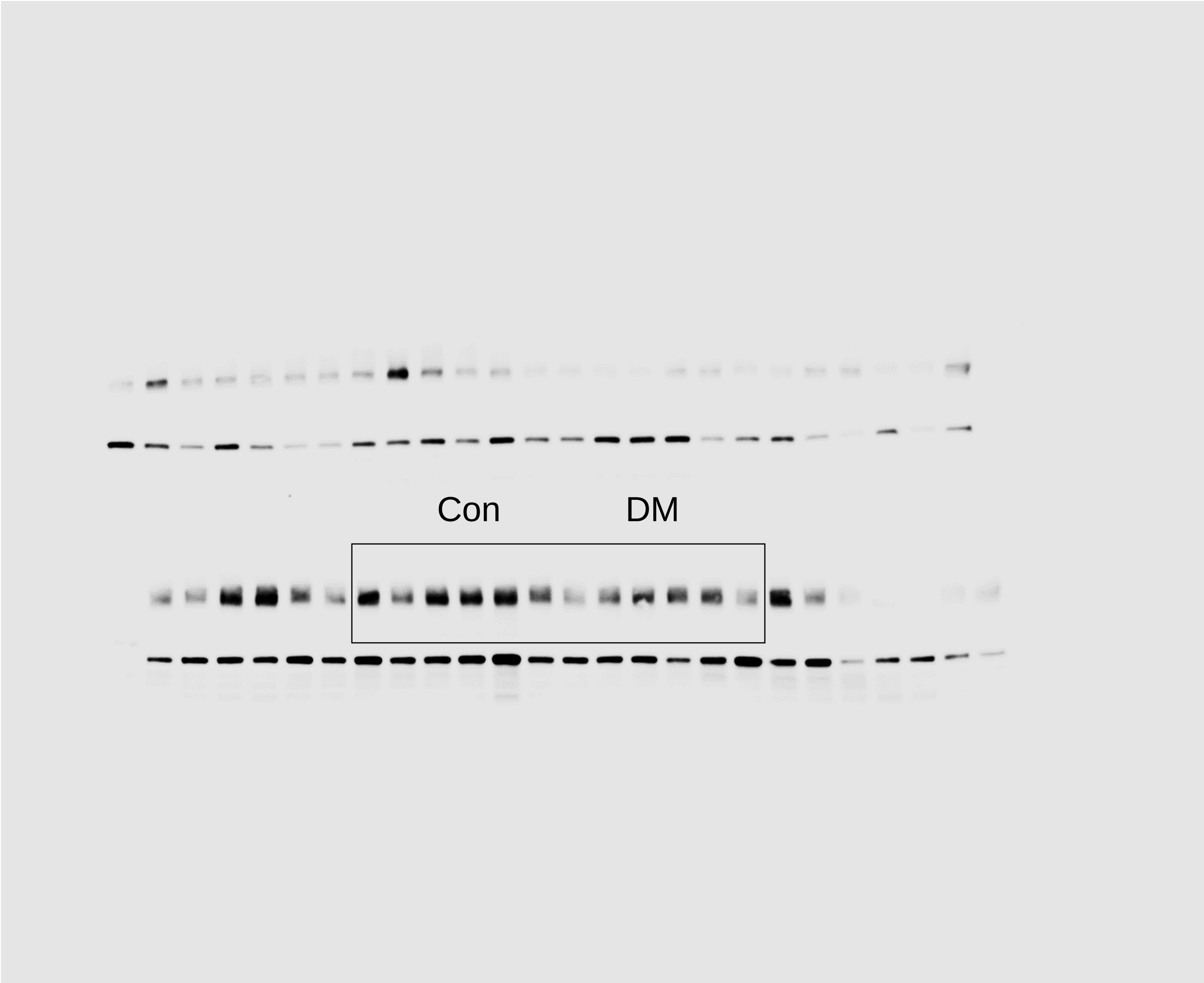

β-actin

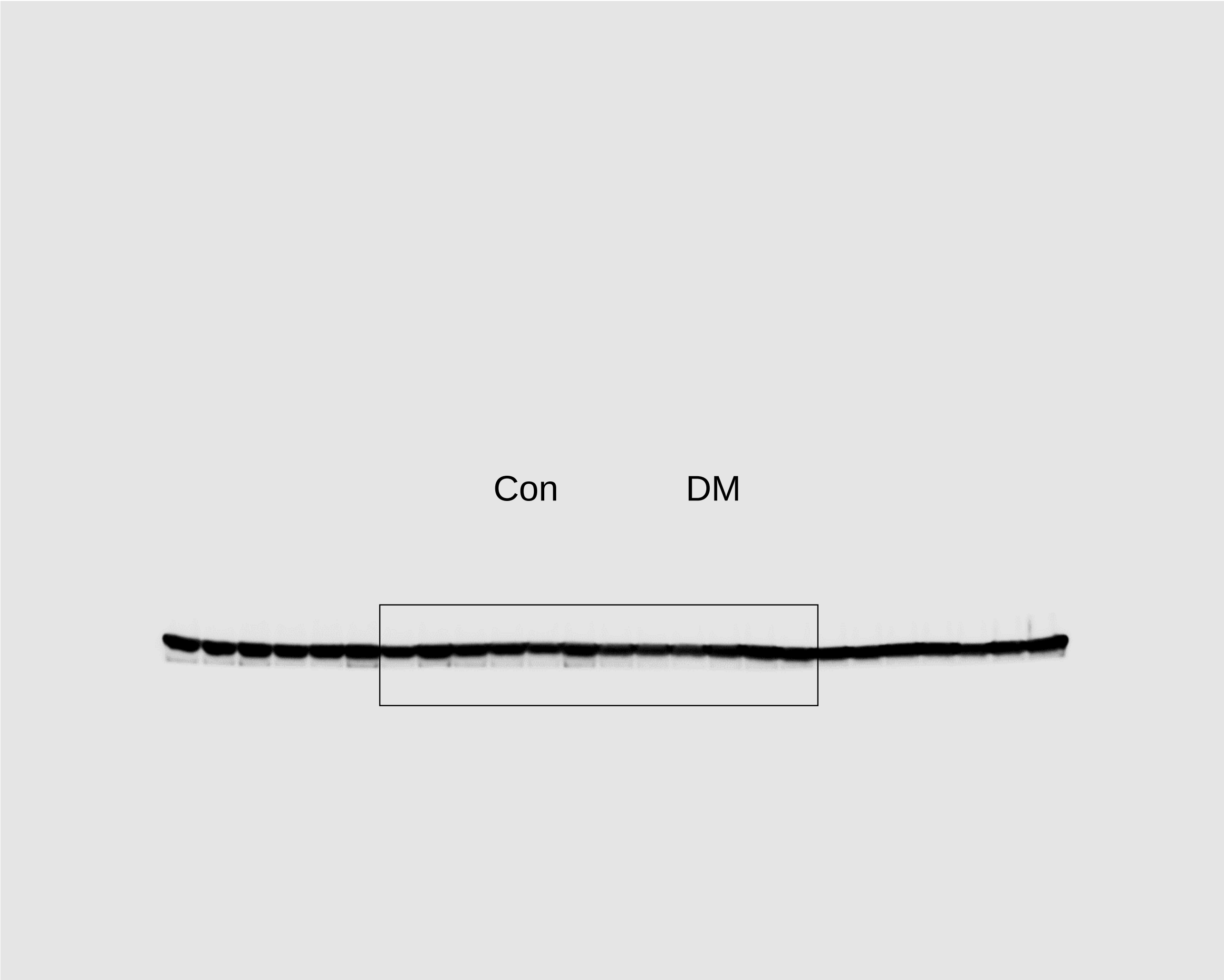

Full-length blots for **Fig. 4** A.

H3K27ac

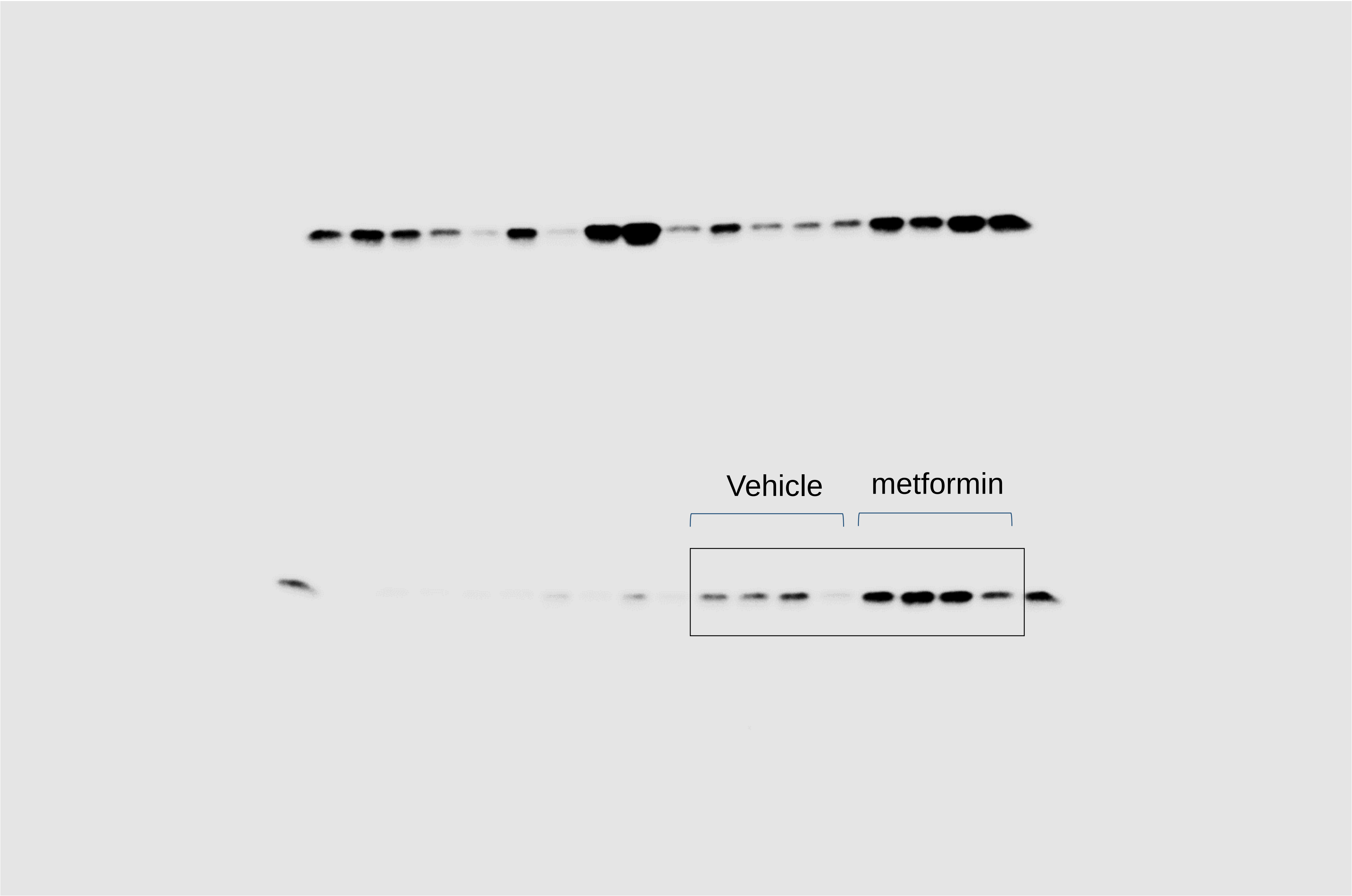

β-actin

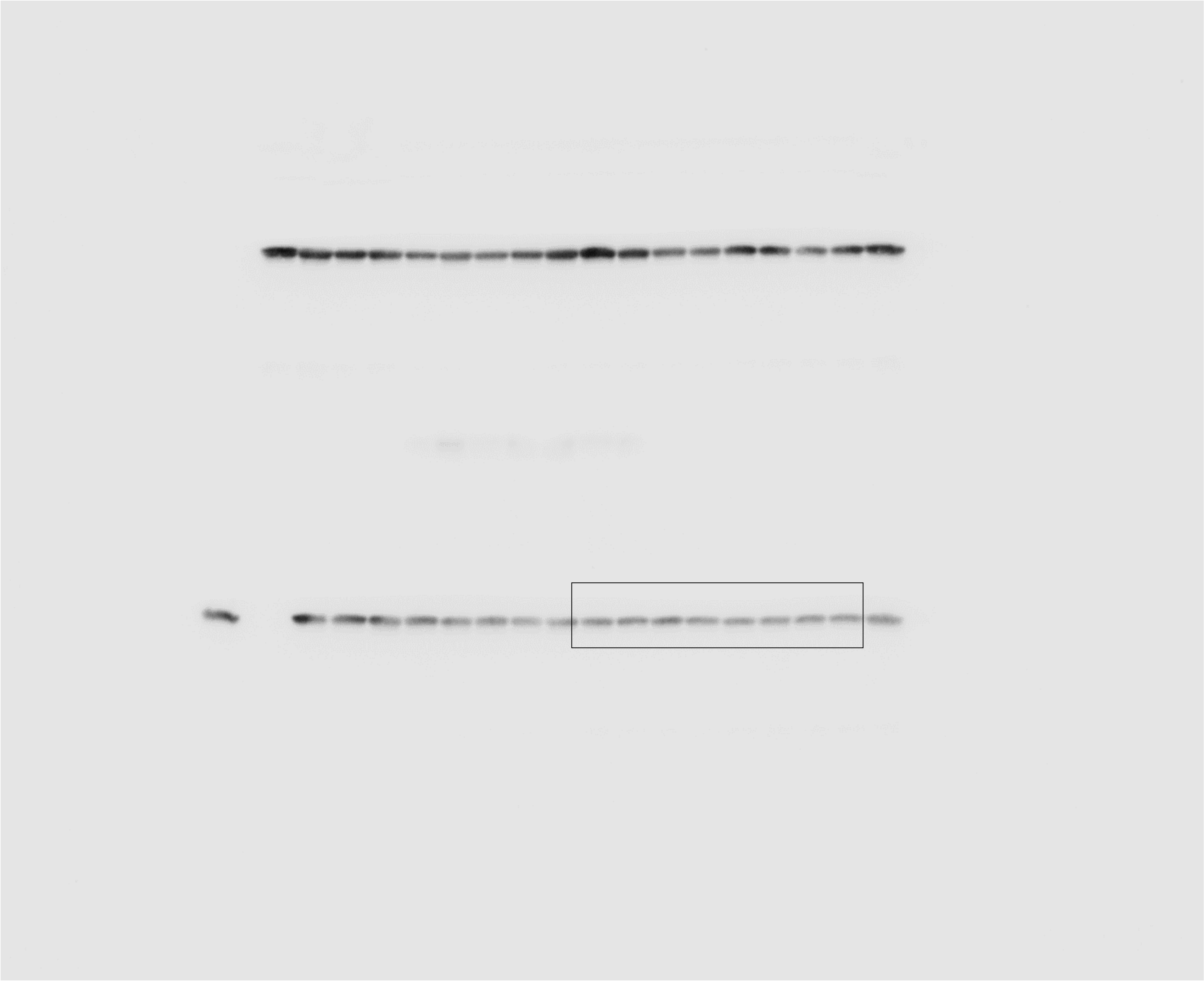

Full-length blots
for **Fig. 4** B.

DNMT1

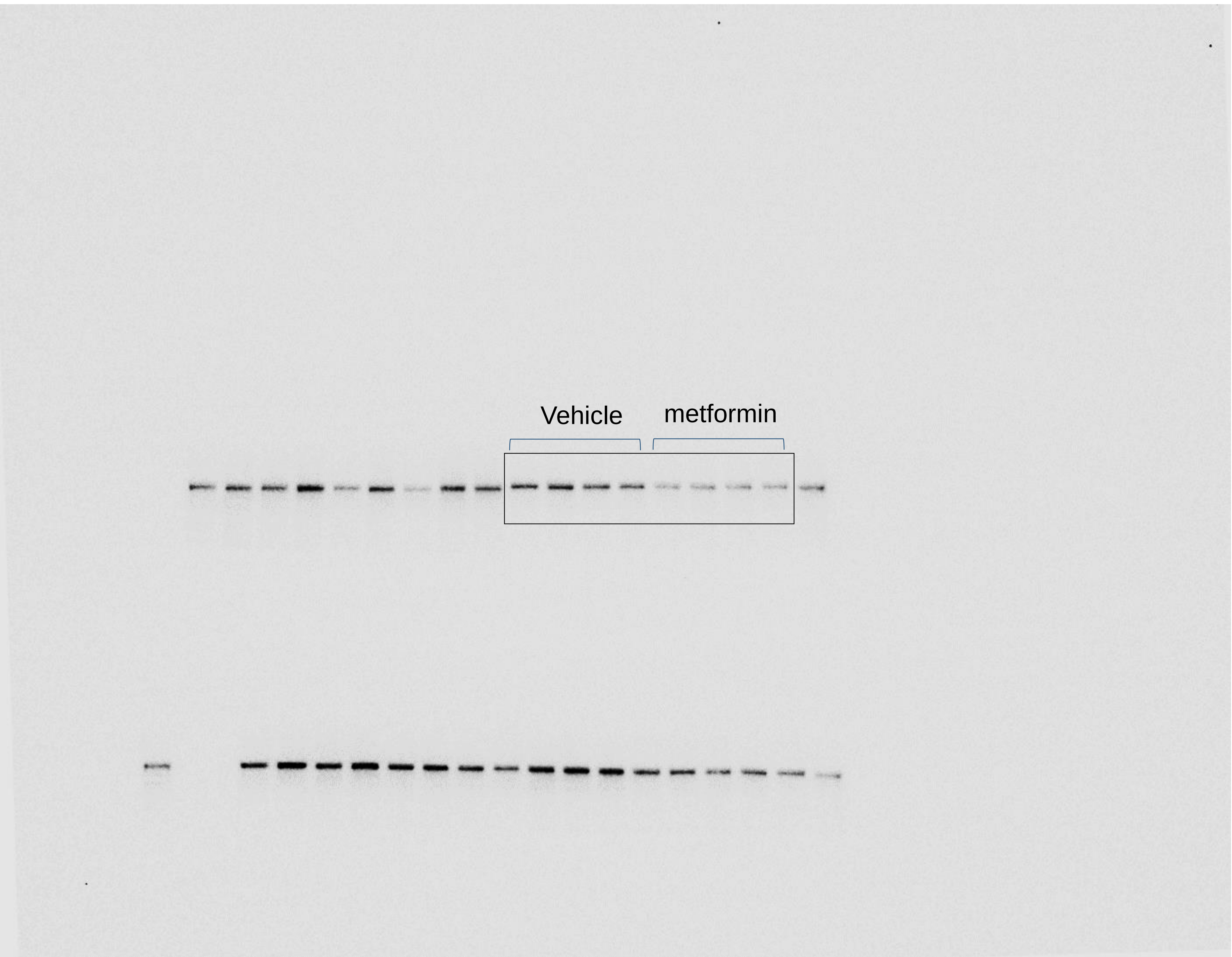

β-actin

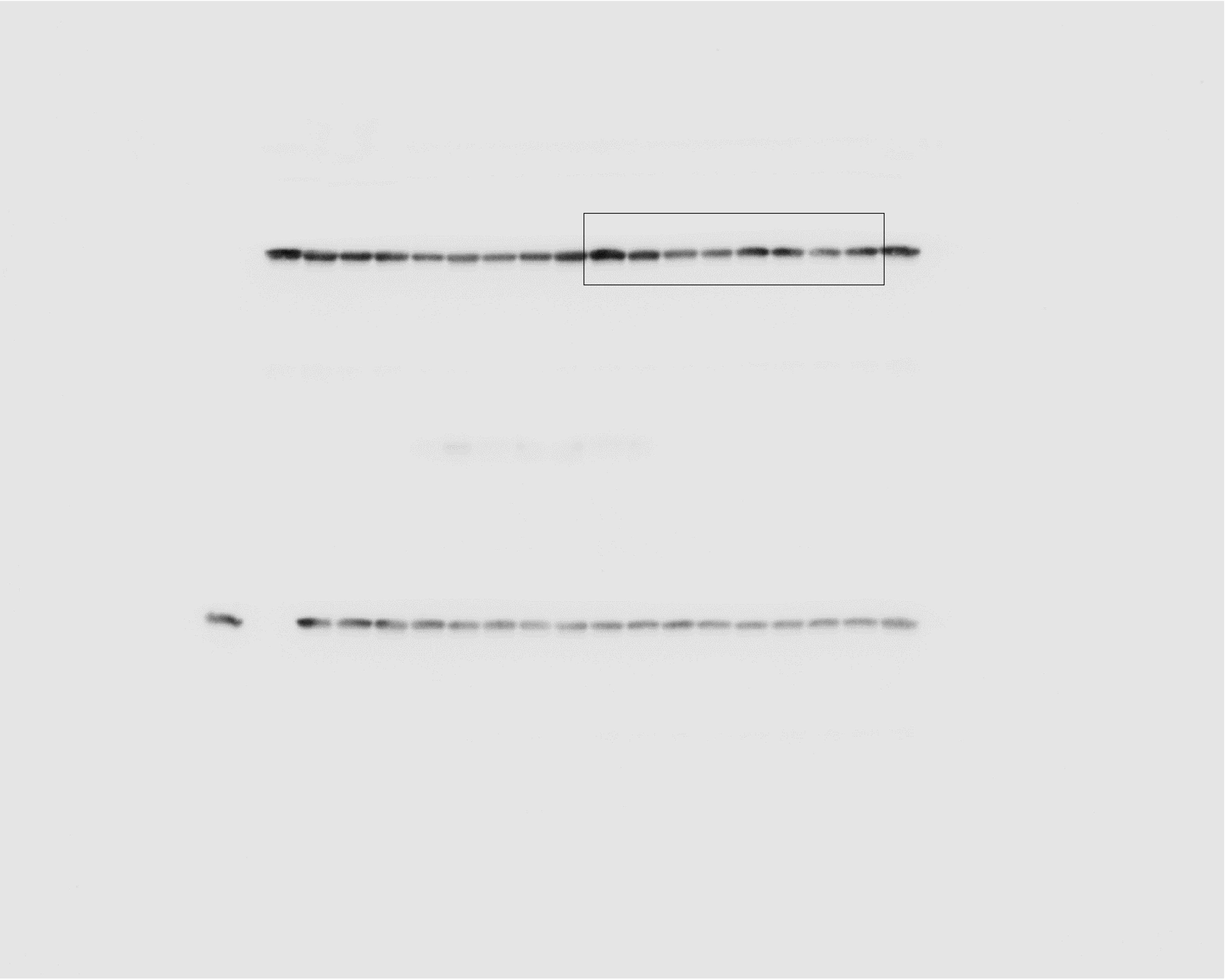

Full-length blots for **Fig. 4**

E.

P-ACC

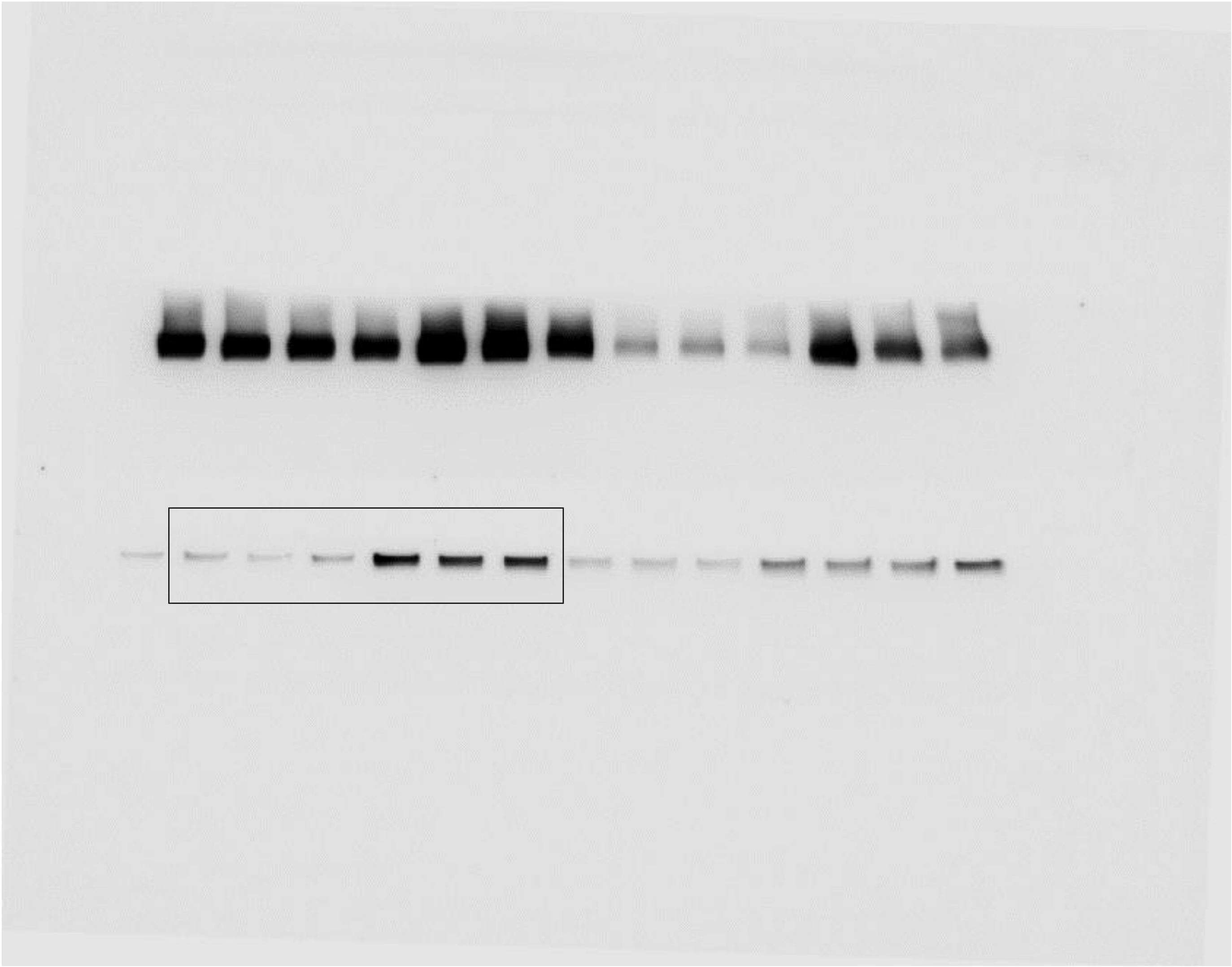

P-AMPKα

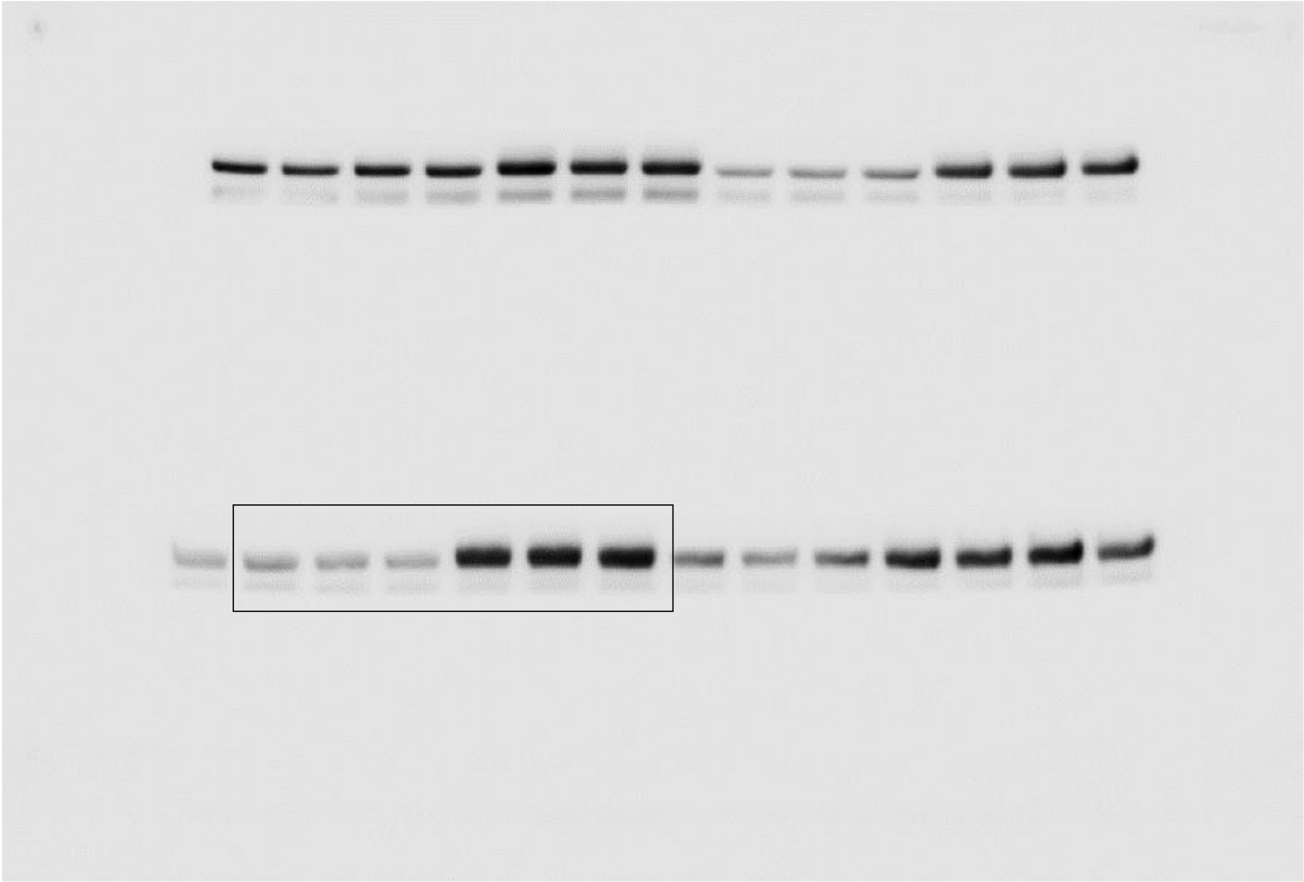

β-actin

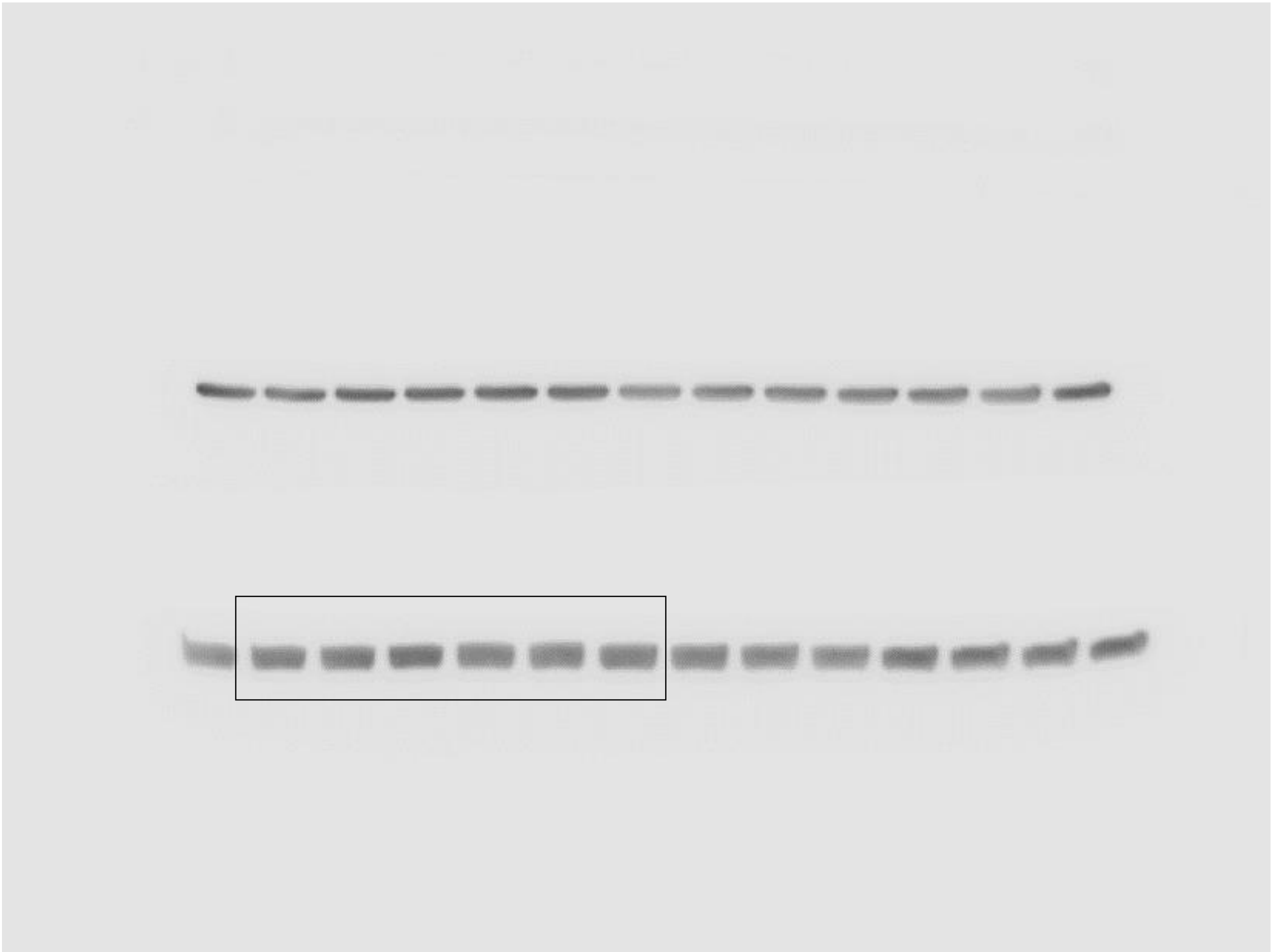

Supplement: Supplementary file 1 — Supplementary information. [file 41598_2020_65415_MOESM1_ESM.pdf]
